# Real Life Experiences as Head of Science

**DOI:** 10.2196/43820

**Published:** 2023-01-09

**Authors:** Jennifer Huberty

**Affiliations:** 1 Fit Minded LLC Phoenix, AZ United States; 2 Mays Cancer Center UT Health San Antonio MD Anderson San Antonio, TX United States

**Keywords:** industry, digital health, business, digital health, technology, science, research, CEO, industry, founder, growth

## Abstract

As the field of digital health grows and evolves, there is a critical need for evidence and theory-based interventions in health care. The need for integration of science into business is more critical than ever. To develop sustainable and scalable products, companies need scientists who understand the industry, can develop scientific strategy that aligns with business priorities, and are able to apply science cross-functionally. In this article, I share the insights I have gained about the roles and responsibilities of industry scientists and the lessons I have learned after spending 5 years as the head of science for a digital health start-up that turned unicorn.

## Introduction

The field of digital health is growing and evolving rapidly, and the current market (ie, wise and skeptical customers, medical community paying attention to new tools, and advancements of regulatory bodies to ensure user safety) demands evidence and theory-based interventions in health care [1,2]. Thus, the need for scientists who can integrate science in business is more critical than ever [3,4]. To develop sustainable and scalable products, companies need scientists who can navigate the industry, develop scientific strategy that aligns with business priorities, and apply science cross-functionally. To date, there have been limited opportunities for researchers to gain relevant training for conducting science in industry. Academia and industry operate very differently, both wanting to develop solutions for better health but with different timelines, priorities, and incentives [5]. For example, in academia, developments are often theory-based and driven by meticulously designed research in carefully controlled settings to establish evidence of effectiveness. In contrast, in industry, development happens rapidly and iteratively, driven primarily by the market, often without theoretical foundations and with little to no supporting evidence [5].

Castro-Sweet and colleagues [4] recently published recommendations to better prepare scientists for meaningful careers in the industry. These included suggestions to network between trainee and industry; facilitate industry scientists as guest speakers for courses, seminars, or the like within academic settings; and find ways to shift the culture in academia to normalize industry positions [4]. However, there is also a distinct lack of resources, discussion, and established best practices on how to integrate science into business. More systematic sharing of real-world experiences and lessons learned would be advantageous to both the scientists who are currently working, or will work, in industry, and the companies that employ them. Below I share the insights I have gained about the roles and responsibilities of industry scientists and the lessons I have learned after spending 5 years as the head of science for a digital health start-up that turned unicorn.

## Roles and Responsibilities

One of the most important roles as a scientist in digital health is the ability to develop scientific strategy aligned with the company’s business priorities. This consideration is quite similar to how scientists often align their research with federal funding priorities. The science strategy must ensure that the company has the evidence and support to market, sell, and/or partner in any way that is related to company goals and how the company wants to grow. As such, initially, a company would utilize data that are already available to them and then report the data (eg, publications) to support their claims. Following what is learned, the science would be developed with more sophisticated designs that will produce more rigorous evidence that is important for supporting the company’s mission. Furthermore, in addition to supporting such missions, industry-based scientists have unique opportunities to continue to advance science in their respective fields owing to the novel data they have access to while working in industry.

Often in industry, science is viewed as a randomized controlled trial or something that is very time and resource intensive. It is the role of the industry scientist to teach the company about other ways in which science can be used to help them. For example, cross-sectional surveys, validated psychological measures, and other tools based on behavior change models can be used to help companies learn more about their customers, their customers’ problems, and if their products are helping solve those problems. Companies generally have a mission and a vision to help their customer with a specific need, whereas the customer may be using their product for a different need, or the product may be helping the customer a way in which they did not expect. These data, collected through surveys and especially when aligned with engagement data, can help with marketing, sales and informing product. Additionally, the data can be published to enhance visibility for the company.

Industry-based scientists must also understand what it means to work cross-functionally (ie, across teams internal to the company, such as product, content, and marketing), and they must be able to execute and communicate science to these different audiences. A scientist must understand what role the other teams play in the company and how science might optimally support those teams’ goals and objectives. Science should enhance roadmaps of other teams, while aiming to minimize additional work. Examples of cross-functional work includes developing cross-sectional surveys with marketing teams to learn more about the customer, working with membership to recruit participants (eg, current users), conducting brief educational sessions for the product and content teams about behavior change and evidence-based strategies for behavior change, developing external partnerships with universities and medical centers to conduct research, and guiding sales teams on how to use science to sell products. 

It is also necessary for the scientist to understand institutional review board (IRB) processes and be very comfortable communicating with commercial IRBs. How data are used and shared and who may need IRB approval will vary with each situation. Clear communication and clarification when needed from a commercial IRB is an essential role of a scientist in industry.

Other responsibilities that are important for an industry scientist in more of a leadership role are building and leading a scientific advisory board (SAB); developing a process for external investigator proposals or partnerships that includes a way to bring revenue into the company; writing and communicating about scientific papers and grants; and building an internal system to conduct rigorous, ethical science as the company grows. Overall, an industry scientist must think with half a business brain and half a science brain; they must understand how to design and implement science that provides evidence for the company, and simultaneously contribute to scientific knowledge. 

## Lessons Learned

Here I address some of the most important lessons I learned in a leadership role as head of science at a digital health start-up. First, the selection of the SAB matters. It is important to vet and engage leading scientists who are passionate about the industry in which they are working. More importantly, the scientists that serve on the SAB should be willing to do the work to help grow the company's scientific agenda. Many times, companies use SABs to showcase that they are working with *big names* in science, but in reality, the SAB members are only attending monthly meetings and do not make meaningful contribution(s) to the company’s scientific priorities. If an SAB is established with very active members that contribute to manuscripts, networking, advising external partners, and grant writing (if applicable), there is bound to be faster growth of science. 

Second, scientists working in industry must be comfortable with change. Digital health start-ups are constantly reinventing themselves. They have business goals to meet, as well as investors with expectations to deal with. For example, a scientist could be working on research aligned with the current month’s priority and find out halfway through data collection that the company is shifting priorities, which means the direction of research needs to be flexible, with a possibility to reset to align with new priorities, deliverables, and timelines. Scientists must also be able to answer perpetual questions, for example: *When will the data be ready? When can we share this with the press?* Therefore, scientists must make sure there are both short- and long-term projects and results that they can generate.

Third, one of the best things about being a scientist in industry who dreams of being funded by the National Institutes of Health (NIH) is that this dream can still be realized. The NIH does fund for-profit companies, and there are also ways for industry to partner with academics and receive funds to conduct collaborative research. Getting NIH funded for conducting research in industry is also beneficial for companies who can use granting success for marketing, partnerships, and sales. 

Fourth, science can enhance business growth, providing a competitive edge. This is especially true for companies operating in business to business and health care markets. For companies, health care organizations, and payers to invest in a behavioral and/or digital health product, they must feel confident that the product works and that it will save money in the long term. Therefore, it is essential to obtain scientific evidence. As head of science, I have seen many sales and partnerships happen because the company was able to demonstrate published evidence for the impact and cost-effectiveness of its product. 

Finally, convincing CEOs and investors (and the rest of the company) of the value of science can sometimes be challenging. It is difficult to calculate the direct return of science for a company. For example, when a paper is published, there are costs to the company, including scientists’ time, IRB approval, publication processing fees, and so on. The return on that investment is not direct revenue but rather the paper provides visibility and validation of the value of the product. This generates leads for sales or partnerships, and eventually leads to increased revenue. Overall, science is versatile and contributes to revenue generation through marketing and press coverage, sales, and partnerships, but a scientist must be able to communicate that to leadership in order for these contributions to be fully appreciated. 

The integration of science into industry is a nascent concept. Academic and industry environments operate differently, and a scientist must be both science- and business savvy. Training is important and, hopefully, as the supply and demand for trained scientists in digital health and health care continues to grow, training for science in industry will improve. In the meantime, learning from industry-based scientists with real-life experiences can help new scientists learn to shift the way they think and be effective at integrating science into business. 

## Abbreviations

IRB: institutional review board
NIH: National Institutes of Health
SAB: scientific advisory board
